# Suspected Suicide Attempts by Self-Poisoning Among Persons Aged 10–19 Years During the COVID-19 Pandemic — United States, 2020–2022

**DOI:** 10.15585/mmwr.mm7216a3

**Published:** 2023-04-21

**Authors:** Rita Farah, Saumitra V. Rege, Ryan J. Cole, Christopher P. Holstege

**Affiliations:** 1 Division of Medical Toxicology, Department of Emergency Medicine, University of Virginia School of Medicine, Charlottesville, Virginia.

The World Health Organization declared COVID-19 a global pandemic on March 11, 2020 (*1*). As strategies to mitigate the pandemic were implemented, concerns were raised that the containment efforts through quarantine and social distancing practices were negatively affecting the mental and physical health of children and adolescents (*2*). Suicide is a growing public health problem in the United States. In 2020, suicide was the second leading cause of death among persons aged 10–14 years and the third leading cause among those aged 15–24 years (*3*). The National Poison Data System (NPDS) database was used to examine trends in suspected suicide attempts by self-poisoning among persons aged 10–19 years before and during the COVID-19 pandemic. Compared with 2019 (prepandemic), during 2021, the overall rate of suspected suicide attempts by self-poisoning increased by 30.0% (95% CI = 28.6%–30.9%), rates among children aged 10–12 years, adolescents aged 13–15 years, and females increased 73.0% (67.4%–80.0%), 48.8% (46.7%–50.9%), and 36.8% (35.4%–38.2%), respectively, and these trends continued into the third quarter of 2022. Substances most frequently involved in overdoses were acetaminophen, ibuprofen, sertraline, fluoxetine, and diphenhydramine. Acetaminophen-involved overdoses increased 71% (67.4%–74.9%) in 2021 and 58.0% (54.5%–61.6%) in 2022. Diphenhydramine-involved overdoses increased 24.2% (19.9%–28.7%) in 2021 and 35.8% (31.2%–40.5%) in 2022. A comprehensive public health approach to suicide prevention, focused on children and adolescents and involving a partnership between families, school teachers, mental health professionals, and public health leadership is needed. The 9-8-8 Suicide and Crisis Lifeline provides crisis support for persons experiencing mental health–related distress and assists community members who are concerned about persons experiencing a mental health crisis.*

A retrospective review of the NPDS database, the data warehouse for all 55 U.S. poison control centers (PCCs), during January 1, 2016–September 30, 2022, was conducted. Each PCC submits, in near real-time, deidentified case data to NPDS after providing necessary poison exposure management and information services to callers from the general public and health care providers. Closed cases coded by specialists in poison information as intentional suspected suicide involving persons aged 10–19 years were included. The NPDS coding manual specifies that cases coded as intentional suspected suicide include suspected suicide attempts as well as intentional self-harm cases. Multiple substances (multiple exposures) can be reported for each call. Cases classified as a confirmed nonexposure (reliable and objective evidence that exposure to a pharmaceutical or nonpharmaceutical agent never occurred) and those involving persons of unknown age were excluded. Reported numeric age was used to manually compute three age group categories (10–12, 13–15, and 16–19 years). Trends in the frequency and rates of suspected suicide attempts by self-poisoning (suspected suicide attempts per 100,000 persons aged 10–19 years) (*4*) were analyzed using Poisson regression methods and stratified by age group, sex, source of calls made to PCCs, level of care, substance involved, and clinical outcome. To assess the potential impact of the pandemic, yearly changes in suspected suicide attempt rates were compared between 2016 and 2022. The year 2019, the last calendar year before the pandemic, was considered the reference year. Monthly trends in suspected suicide attempt calls were plotted for January 2019–September 2022. Monthly counts of suspected suicide attempts and overall human exposure calls^† ^during the pandemic were compared with their corresponding 2019 reference months. Interpretation of trends in suspected suicide attempts considered the potential impact of changes in the monthly overall human exposure calls to PCCs. Data were analyzed using SAS statistical software (version 9.4; SAS Institute). Percent changes are reported with the corresponding 95% CIs. The study was conducted on deidentified NPDS data and was exempt from comprehensive Institutional Review Board review.

The yearly trend of suspected suicide attempt rates showed a sharp increase in 2021 compared with previous years. From 2019 to 2021, the overall number of human exposure calls to PCCs decreased 3.1%, from 2,148,141 to 2,080,917 (p<0.001); however, suspected suicide attempt calls increased (29.5%) in 2021 compared with prepandemic rates (2019). Calls to PCCs regarding suspected suicide attempts increased for both males and females (except between 2018 and 2019) and across all age groups (Table). In 2021, a statistically significant increase in the rate of suspected suicide attempts occurred among children aged 10–12 years (73.0%), adolescents aged 13–15 years (48.8%), and females (36.8%); this increase continued into September 2022. Call rates made to PCCs from health care facilities (29.0%) and the general public (33.0%) increased during 2021 as did admissions to psychiatric facilities (29.3%). 

**TABLE:** Frequency and rates* of suspected suicide attempts by self-poisoning among persons aged 10–19 years reported to U.S. poison control centers, by selected characteristics — National Poison Data System, United States, January 1, 2016–September 30, 2022

Characteristic	No.^† ^(rate)							% Rate change (95% CI)^†^					
Year	2016	2017	2018	2019	2020	2021	2022	2016	2017	2018	2020	2021	2022^†^
**Overall**	**79,984^§^** **(189.3)**	**87,553^§^** **(206.7) **	**88,227^§^ (208.1) **	**88,535** **(209.2) **	**93,265^§^** **(220.7) **	**114,664** **(272.0) **	**104,888^§^** **(248.6) **	** −10.1 (−10.5 to −8.8)^§^**	**−1.2 (−3.0 to −0.2)^§^**	**−0.5 (−1.4 to 0.5)**	**5.4 (4.4 to 6.4)^§^**	**30.0 (28.6 to 30.9)^§^**	**18.6 (17.7 to 19.7)^§^**
**Age group, yrs**													
10–12	3,450^§^ (27.5)	4,286^§^ (33.9)	4,951^§^ (39.0)	5,473 (43.4)	6,423^§^ (51.5)	9,396^§^ (76.1)	8,171^§^ (66.1)	−37.0 (−39.6 to −34.2)^§^	−21.9 (−24.9 to −18.7)^§^	−10.0 (−13.3 to 6.4)^§^	17.8 (13.6 to 22.1)^§^	73.0 (67.4 to 80.0)^§^	50.4 (45.4 to 55.7)^§^
13–15	30,572^§^ (243.5)	33,207^§^ (265.0)	33,278^§^ (264.2)	34,007 (268.8)	38,614^§^ (303.4)	50,844^§^ (397.6)	45,944^§^ (361.6)	−9.8 (−11.2 to −8.4)^§^	−2.0 (−3.4 to −0.5)^§^	−2.0 (−3.5 to −0.5)^§^	13.2 (11.6 to 14.9)^§^	48.8 (46.7 to 50.9)^§^	34.9 (33.0 to 36.8)^§^
16–19	45,810^§^ (267.2)	49,914^§^ (290.3)	49,848^§^ (291.0)	48,902 (286.4)	48,075^§^ (282.1)	54,249^§^ (318.7)	50,617^§^ (295.7)	−6.5 (−7.3 to −5.3)^§^	1.8 (0.5 to 3.0)^§^	1.8 (0.5 to 3.1)^§^	−1.7 (−2.9 to −0.4)^§^	11.1 (9.7 to 12.4)^§^	3.4 (2.1 to 4.7)^§^
**Sex**													
Female	62,915^§^ (303.3)	68,995^§^ (332.9)	67,944 (327.4)	68,045 (328.5)	73,270^§^ (354.2)	92,962^§^ (450.1)	83,131^§^ (402.6)	−7.6 (−8.4 to −6.6)^§^	1.4 (0.3 to 0.8)^§^	−0.3 (−1.3 to 0.8)	7.7 (6.6 to 8.9)^§^	36.8 (35.4 to 38.2)^§^	22.3 (21.1 to 23.6)^§^
Male	16,969^§^ (78.9)	18,452^§^ (85.3)	20,174 (93.2)	20,362 (94.3)	19,848^§^ (92.0)	21,467^§^ (99.7)	21,480^§^ (99.7)	−16.6 (−8.2 to −14.8)^§^	−9.5 (−11.3 to −7.7)^§^	−1.1 (−3.0 to 0.9)	−2.5 (−4.4 to −0.6)^§^	5.6 (3.6 to 7.6)^§^	5.6 (3.6 to 9.4)^§^
**Source of call**													
Health care facilities	64,137^§^ (151.7)	70,986 (167.6)	71,690^§^ (169.0)	71,061 (167.9)	73,175^§^ (173.2)	91,522^§^ (216.9)	84,484^§^ (200.3)	−9.7 (−10.7 to −8.2)^§^	−0.2 (−1.2 to 0.9)	0.8 (−0.3 to 1.8)	3.0 (2.0 to 4.1)^§^	29.0 (27.7 to 30.3)	19.0 (17.7 to 20.2)
General public	15,688^§^ (37.1)	16,390^§^ (38.7)	16,397^§^ (38.7)	17,321 (40.9)	19,939^§^ (47.2)	22,997^§^ (54.5)	20,288^§^ (48.1)	−9.4 (−11.4 to −7.5)^§^	−5.5 (−7.5 to −3.4)^§^	−5.5 (−7.5 to −3.4)^§^	15.2 (12.7 to 17.6)^§^	33.0 (30.4 to 35.6)^§^	17.3 (14.9 to 19.7)^§^
**Level of care**													
Admission to critical care unit	13,632^§^ (32.3)	14,389^§^ (34.0)	13,655^§^ (32.2)	13,095 (30.9)	13,105 (31.0)	14,114^§^ (33.4)	11,879^§^ (28.0)	4.2 (1.6 to 6.7)^§^	9.8 (7.3 to 12.5)^§^	4.2 (1.7 to 6.7)^§^	0.1 (−0.3 to 2.6)	8.0 (5.4 to 10.6)^§^	−9.2 (−11.4 to −6.9)^§^
Admission to psychiatric facility	25,186^§^ (59.6)	28,515^§^ (67.3)	29,771^§^ (70.2)	30,485 (72.0)	31,636^§^ (74.9)	39,354^§^ (93.3)	35,924^§^ (85.2)	−17.4 (−18.8 to −16.0)^§^	−6.6 (−8.1 to −5.0)^§^	−2.5 (−4.0 to −0.9)^§^	3.8 (2.2 to 5.5)^§^	29.3 (27.3 to 31.2)^§^	18.0 (16.2 to 19.9)^§^
**Clinical outcome**													
Major effect^¶^	1,919^§^ (4.5)	2,269^§^ (5.3)	2,545^§^ (6.0)	2,980^§^ (7.4)	3,139^§^ (7.4)	3,757^§^ (8.9)	3,501^§^ (8.3)	−35.7 (−39.3 to −31.9)^§^	−14.0 (−28.0 to −19.7)^§^	−14.8 (−19.2 to −10.2)^§^	5.3 (0.2 to 10.7)^§^	26.1 (20.2 to 32.4)^§^	17.6 (12.0 to 23.4)^§^
Death**	37^§^ (0.9)	33^§^ (0.8)	51 (0.12)	59 (0.14)	55 (0.13)	65 (0.15)	59 (0.14)	−37.3 (−59.4 to −42.4)^§^	−44.1 (−63.5 to −14.4)^§^	13.7 (−40.7 to 25.6)	−6.8 (−35.6 to 34.7)	10.3 (−22.4 to 57.0)	0.1 (−30.2 to 43.6)
**Substance^††^**													
Acetaminophen	10,178^§^ (24.1)	11,397^§^ (26.9)	11,709^§^ (27.6)	12,552 (29.6)	15,141^§^ (35.8)	21,443^§^ (50.9)	19,805^§^ (46.8)	−18.9 (−21.0 to −16.8)^§^	−9.3 (−11.6 to −7.0)^§^	−6.9 (−9.3 to −4.5)^§^	20.7 (17.9 to 23.6)^§^	71.0 (67.4 to 74.9)^ §^	58.0 (54.5 to 61.6)^§^
Ibuprofen	11,851^§^ (28.0)	13,015^§^ (30.7)	13,162^§^ (31.0)	13,353 (31.5)	13,756^§^ (32.5)	18,017^§^ (42.7)	15,513^§^ (36.7)	−11.3 (−13.4 to −9.0)^§^	−2.6 (−4.9 to −0.2)^§^	−1.6 (−3.9 to −0.9)^§^	3.1 (0.7 to 5.6)^§^	35.1 (32.1 to 38.2)^§^	16.3 (13.6 to 19.0)^§^
Sertraline	4,366^§^ (10.3)	5,079^§^ (12.0)	5,488^§^ (12.9)	5,791 (13.7)	6,394^§^ (15.1)	7,651^§^ (18.1)	6,896^§^ (16.3)	−24.6 (−27.5 to −21.6)^§^	−12.4 (−15.4 to −9.0)^§^	−5.4 (−8.8 to −1.8)^§^	10.5 (6.6 to 14.5)^§^	32.3 (27.9 to 36.9)^§^	19.2 (15.1 to 23.5)^§^
Fluoxetine	3,935^§^ (9.3)	4,518^§^ (10.7)	4,943^§^ (11.7)	592^§^ (12.5)	5,698^§^ (13.5)	7,620^§^ (18.1)	6,812^§^ (16.1)	−25.7 (−28.7 to −22.5)^§^	−14.7 (−18.0 to −11.3)^§^	−6.7 (−10.3 to −3.0)^§^	7.7 (3.8 to 11.9)^§^	44.2 (39.2 to 49.4)^§^	28.9 (24.3 to 33.6)^§^
Diphenhydramine	5,191^§^ (12.3)	5,771^§^ (13.6)	5,551^§^ (13.1)	5,596 (13.2)	5,864^§^ (13.8)	6,940^§^ (16.5)	7,587^§^ (18.0)	−7.2 (−10.7 to −3.7)^§^	3.1 (−0.7 to 6.9)	−0.1 (−4.6 to 2.8)	4.8 (1.1 to 8.8)^§^	24.2 (19.9 to 28.7)^§^	35.8 (31.2 to 40.5)^§^

* Attempts per 100,000 population based on U.S. Census Bureau’s midyear census.

^†^ Annualized rates were used to present data during January 1, 2019–September 30, 2022 (reference year: 2019).

^§^ Statistically significant.

^¶^ Symptoms resulting from exposures that were life-threatening or resulted in significant residual disability or disfigurement.

** Direct and indirect death reports were included.

^††^ Generic codes: acetaminophen alone (0072705, 0072707, and 0072000), ibuprofen (0234003), sertraline (310014), fluoxetine (0310011), and diphenhydramine alone (0159900, 0159000, and 0159850).

In 2021 and 2022, an analysis of substances involved in suspected suicide attempts found acetaminophen, ibuprofen, sertraline, fluoxetine, and diphenhydramine to be the substances most frequently involved, with a significant increase in acetaminophen- (71.0% and 58.0%, respectively) and diphenhydramine- (24.2% and 35.8%, respectively) involved overdoses compared with those in 2019. Overdoses involving ibuprofen (35.1%), fluoxetine (44.2%), and sertraline (32.3%) increased significantly in 2021 compared with prepandemic rates (2019) (Figure 1). Single-substance cases accounted for 430,051 (68%) suspected suicide attempt calls; acetaminophen (excluding combinations with other substances) was the most frequent single substance involved in suspected suicide attempts, accounting for 57,768 (13.4%) of single-substance cases.

**FIGURE 1:**
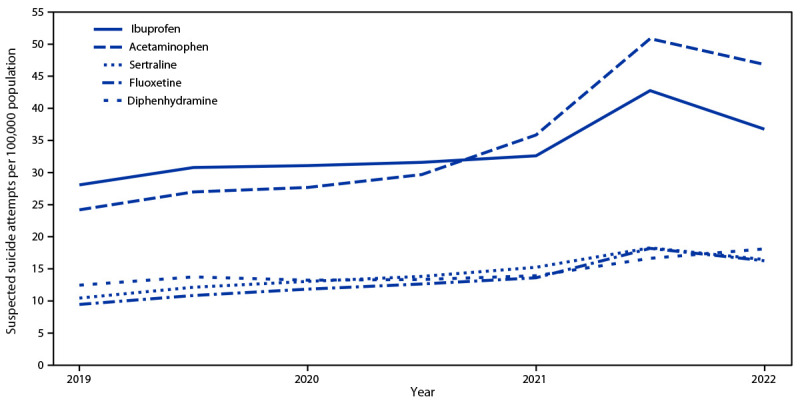
Rates of suspected suicide attempts by self-poisoning among persons aged 10–19 years reported to U.S. poison control centers, by substance*^,^^†^ — United States, January 1, 2016–September 30, 2022^§^ * Trends in rates of suspected suicide attempts involving acetaminophen, ibuprofen, sertraline, and fluoxetine were statistically significant during 2016–2022 (reference year: 2019). ^†^ Trends in rates of suspected suicide attempts involving diphenhydramine were statistically significant during 2020–2022 (reference year: 2019). ^§^ Annualized rates were used to present data during January 1, 2019–September 30, 2022.

The monthly variation in suspected suicide attempt–related calls among persons aged 10–19 years increased during school months, with a sharp increase in September, and a decline during summer months (June and July) and winter breaks (December) (Figure 2). During the period when the national lockdown was implemented (April–May 2020), suspected suicide attempt–related calls were lower compared with those during the same months in 2019, 2021, and 2022 (p<0.001); the overall human exposure calls did not decrease during this same period. However, during June 2020–February 2022, monthly suspected suicide attempt–related calls were significantly higher than they were during the corresponding months of 2019 (with the exception of March–June 2020, when suspected suicide attempt–related calls were similar to those in 2019). The monthly overall human exposure calls to PCCs significantly declined beginning in August 2020 compared with the corresponding months during 2019 (Supplementary Table, https://stacks.cdc.gov/view/cdc/126401). 

**FIGURE 2:**
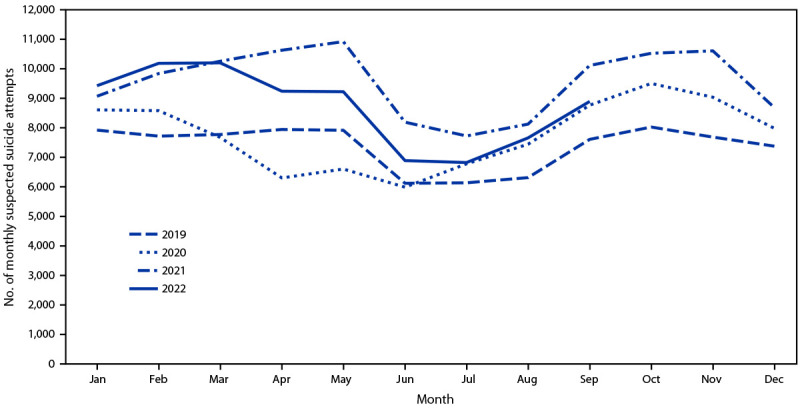
Number of monthly suspected suicide attempts by self-poisoning among persons aged 10–19 years reported to U.S. poison control centers — United States, January 1, 2019–September 30, 2022

## Discussion

This study, examining the potential impact of the COVID-19 pandemic on suspected suicide attempts by self-poisoning among children and adolescents using a U.S. national database, found an increase during the pandemic, most notably among children aged 10–12 years, adolescents aged 13–15 years, and females, with the sharpest increase in attempts involving acetaminophen and diphenhydramine. Further, the study revealed an increase in admissions to psychiatric facilities. These findings suggest that the mental health of children and adolescents was affected by the pandemic, raising concerns about long-term consequences, especially given that previous attempted suicide has been found to be the strongest predictor of subsequent death by suicide (*5*). The results support findings of an increase in emergency department visits for suspected suicide attempts among persons aged 12–17 years, particularly among adolescent girls, beginning in mid-2020, reported in mid-2021, based on data from the National Syndromic Surveillance Program (*6*). An increase in the proportion of suspected suicide-related calls during 2015–2020, involving acetaminophen and ibuprofen, among children aged 10–12 years, was recently reported, in an analysis of calls to PCCs using NPDS data *(**7**)*. The current findings revealed an increase in rates of suspected suicide attempts involving acetaminophen and ibuprofen among U.S. population aged 10–12 years during 2016–2022; however, the increase during the pandemic was at an accelerated rate.

The period coinciding with the beginning of the pandemic and stay-at-home orders (March 2020) was characterized by an initial decrease in reported cases of suspected attempted suicide, followed by a subsequent increase (July 2020). A similar trend in suicides is commonly seen among persons affected by crises such as natural disasters and wars. This phenomenon is frequently termed the “honeymoon effect” *(**8**)*. The seasonality of suicide among children and adolescents is reported in the literature and is characterized by a significant decline during the months of June, July, and August, as well as December (*9*). The seasonality of suspected suicide attempts among youth during the pandemic can be observed starting in September 2020. 

In the current study, rates of acetaminophen-involved suicide attempts in 2020 surpassed those of ibuprofen that had predominated in earlier years and continued to increase through September 2022. Three of the top five most frequently identified drugs involved in suspected suicide attempts in this analysis are over-the-counter medications, and two drugs are antidepressant medications. An urgent need exists to strengthen programs focused on identifying and supporting persons at risk for suicide, especially young persons. In addition, protective environments need to be created through the reduction of access to lethal means including promoting the safe storage of medications (e.g., over-the-counter drugs). 

The findings in this report are subject to at least four limitations. First, NPDS data are not designed to assess potential risk factors leading to increases in suspected suicide attempts. However, visits to an emergency department for mental health consults and suspected child abuse, risk factor for potential suicide attempts, also increased in 2020 compared with 2019 (*10*). Further, call volume to PCCs decreased during the pandemic; therefore, the increase in calls for suspected suicide attempts cannot be explained by the change in call volume. In addition, although the U.S. population aged 10–19 years decreased 0.4% from 42,314,777 in 2019 to 42,190,515 in 2021 (*4*), this decrease cannot fully account for the increase in rates of suspected suicide attempts during the pandemic. Second, because NPDS data are affected by completeness of reporting from health care providers and the general public, as well as the accuracy of data entry and coding by PCC staff members, they are susceptible to reporting bias. Third, multiple substances can be reported for each call to PCCs, and it was not possible to determine which substance was most related to the clinical effects and medical outcome. Finally, because reporting to PCCs is voluntary, NPDS data do not represent all cases of suspected suicide attempts. However, the consistency of these findings with those in other published studies highlights the association of the COVID-19 pandemic with harmful effects on youth mental health and underscores the need for further research to confirm these findings and inform prevention strategies *(**6**,**10**)*.

Pediatric and adolescent suicide attempts by self-poisoning have increased during the COVID-19 pandemic. It is imperative to mitigate this increase with suicide prevention measures that focus on children and adolescents and involve partnerships between key partners in the communities, such as families, school teachers, mental health professionals, and public health leadership. Suicide prevention resources and tools are available to help communities prevent suicide. These strategies include identifying and supporting youth at risk for suicide, creating protective environments through reduction of access to lethal means, improving access to mental health care, and teaching coping and problem-solving skills. In addition, the 9-8-8 Suicide and Crisis Lifeline became available nationally in July 2022. The 9-8-8 Suicide and Crisis Lifeline is a national network of more than 200 crisis centers supported by local and state sources as well as the Department of Health and Human Services’ Substance Abuse and Mental Health Services Administration*.*

**Summary**

**What is already known about this topic?**

In 2020, suicide was the second leading cause of death among persons aged 10–14 years and the third leading cause among those aged 15–24 years.

**What is added by this report?**

Analysis of National Poison Data System data found that the rate of suspected suicide attempts by self-poisoning among persons aged 10–19 years increased 30.0% in 2021 as compared with prepandemic rates (2019), with a 73.0% increase among children aged 10–12 years, 48.8% among adolescents aged 13–15 years, and 36.8% among females.

**What are the implications for public health practice?**

A comprehensive public health approach to suicide prevention measures focusing on children and adolescents and involving partnerships among families, school teachers, mental health professionals, and public health leadership is needed.

## Footnotes

*
https://www.cdc.gov/suicide/prevention/index.html

† Total number of calls made to PCCs regarding actual or suspected human contact with any substance that was ingested, inhaled, absorbed, applied to, or injected into the body, regardless of toxicity or clinical manifestation. PCCs receive calls related to human and animal exposures.
